# Identifying robust hysteresis in networks

**DOI:** 10.1371/journal.pcbi.1006121

**Published:** 2018-04-23

**Authors:** Tomáš Gedeon, Bree Cummins, Shaun Harker, Konstantin Mischaikow

**Affiliations:** 1 Department of Mathematical Sciences, Montana State University, Bozeman, Montana, United States of America; 2 Department of Mathematics, Rutgers, The State University of New Jersey, Piscataway, New Jersey, United States of America; Duke University, UNITED STATES

## Abstract

We present a new modeling and computational tool that computes rigorous summaries of network dynamics over large sets of parameter values. These summaries, organized in a database, can be searched for observed dynamics, e.g., bistability and hysteresis, to discover parameter regimes over which they are supported. We illustrate our approach on several networks underlying the restriction point of the cell cycle in humans and yeast. We rank networks by how robustly they support hysteresis, which is the observed phenotype. We find that the best 6-node human network and the yeast network share similar topology and robustness of hysteresis, in spite of having no homology between the corresponding nodes of the network. Our approach provides a new tool linking network structure and dynamics.

## Author summary

To summarize our understanding of how genes, their products and other cellular actors interact with each other, we often employ networks to describe their interactions. However, networks do not fully specify how the underlying biological system behaves in different conditions, nor how such response evolves in time. We present a new modeling and computational approach that allows us to compute and collect summaries of network dynamics for large sets of parameter values. We can then search these summaries for all observed behavior. We illustrate our approach on networks that govern entry to the cell cycle in humans and yeast. We rank networks based on how robustly they exhibit the experimentally observed behavior of hysteresis. We find similarities in network structure of the best ranked networks in yeast and humans, which are not explained by a common ancestry. Our approach provides a tool linking network structure and the behavior of the underlying system.

## Introduction

In cell biology, the power of a network model as an organizational principle of complex regulation rests on the premise that there is a predictive relationship between the network structure and the network dynamics [1–4]. A network model only requires specifying the character of the interactions between genes, proteins and signaling molecules, which can be inferred with relative ease compared to the parameters governing these interactions. If the premise of a predictive relationship holds, then the network approach to complex regulation is highly advantageous, since the phenotype of the cell encoded in its dynamics can be deduced only from the interaction data.

The firm bridge between network structure and the dynamics of the corresponding nonlinear system remains elusive for the fundamental reason that it cannot exist in the suggested generality. The dynamics will always depend on the state of the cell, which in the models is represented by the parameters and initial data.

Some partial results in terms of motif theory have been suggested [1], but these are limited to small networks and their applicability to the dynamics of larger networks is questionable [5, 6]. Furthermore, there is currently no mathematical theory that suggests that understanding of dynamics of a small motif that is embedded in a larger network informs our knowledge of the dynamics of the larger network. In fact, the classical theory of dynamical systems lacks tools that describe dynamics when parameters are unmeasured, or, if measured, carry large uncertainty.

In this paper we report on a new approach [7–9] referred to as Dynamic Signatures Generated by Regulatory Networks (DSGRN) that provides a queryable global characterization of dynamics over large regions of parameter space. This is based on a new, still developing, computationally efficient perspective of nonlinear dynamics [10–12]. The philosophy of this approach has already seen applications in other settings [13–16]. Novel features of DSGRN include the following: (i) DSGRN does not use an explicit functional form for the nonlinearities governing the dynamics, (ii) the decomposition of parameter space reflects the representation of the nonlinear dynamics, and (iii) the decomposition of parameter space is determined by information local to each node of the regulatory network, and this local determination is computed a priori.

For the sake of clarity we discuss DSGRN in the specific, but important biological context of *resettable bistability* and *hysteresis*, especially as they relate to cell cycle restriction point dynamics.

**Fig 1:**
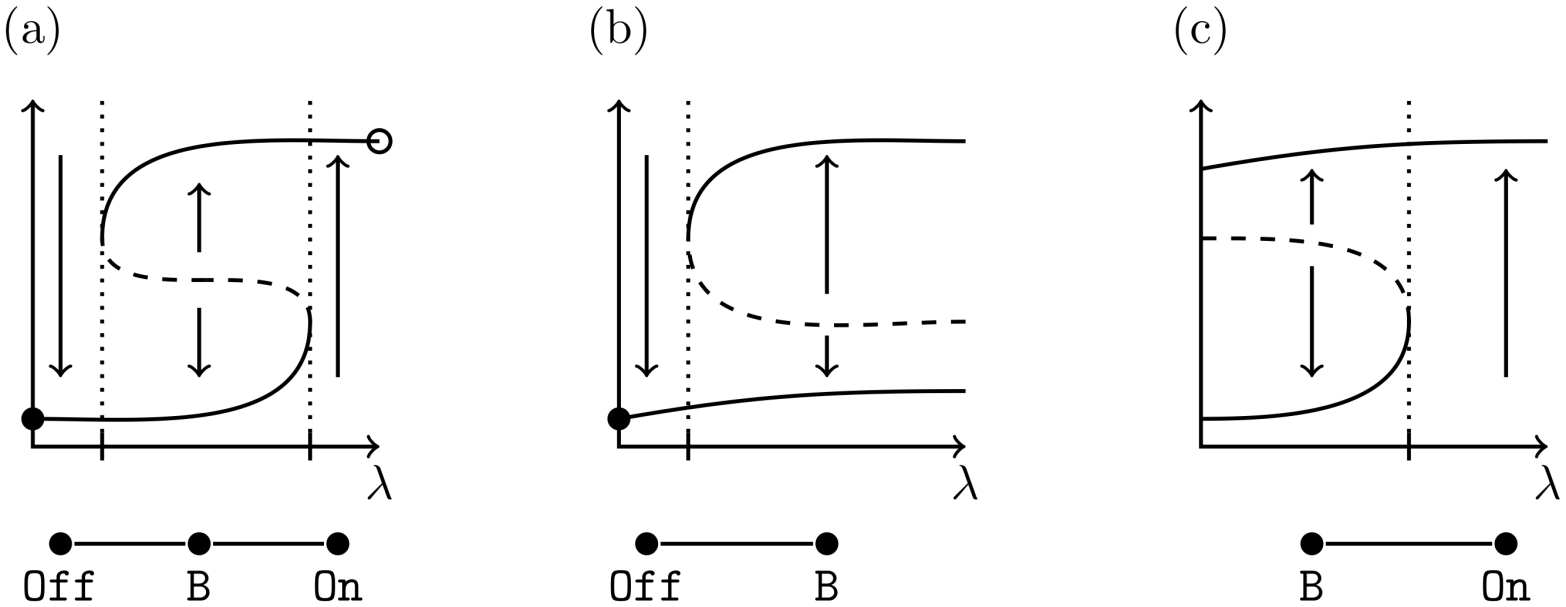
Hysteresis and resettable bistability. Solid (dashed) lines indicate stable (unstable) equilibria for fixed values of input, Off = Low Equilibrium, B = Bistability, and On = High Equilibrium. *Resettable bistability:* When input signal is withdrawn from a bistable system, and the system resets to the low equilibrium (filled circle). *Hysteresis:* In addition to resettable bistability, when signal is increased, system goes to high equilibrium (circle). (a) Hysteresis; (b) resettable bistability, but not hysteresis. (c) No resettable bistability.

A key decision for each cell is when to replicate DNA and initiate proliferation. This decision is based on multiple factors, but once the process has started DNA replication must be finished. Therefore the influence of these factors must be uncoupled at the moment of the decision, called the *restriction point* of the cell cycle [17–19]. The requirement of irreversibility and decoupling suggest that phenotypically a *bistable switch* may underlie the restriction point. The simplest model of a bistable switch involves a hysteresis curve as indicated in Fig 1(a) where the curve indicates the equilibria for a differential equation $\dot{x}=f\left(x,\lambda \right)$ and λ is a parameter. Parameter space naturally divides into three intervals, low λ and high λ for which there exists a single stable fixed point denoted Off and On, respectively, and medium values of λ for which there exist two stable fixed points (B).

Assume the system is in the On state. In the setting of Fig 1(a) or 1(b) if the value of λ is decreased by a sufficient amount (beyond the left hash mark) then the internal dynamics of the system will drive it to the Off state. This is not the case in the setting of Fig 1(c). Observe that the global structure indicated in Fig 1(a) allows for the occurence of hysteresis, i.e. the ability to repeatedly reset the system from On to Off and from Off to On by changing the value of λ and a region of parameter space, the medium values of λ, at which the direction of of the change in λ (increasing or decreasing) determines whether the system is in the on or off state.

**Fig 2:**
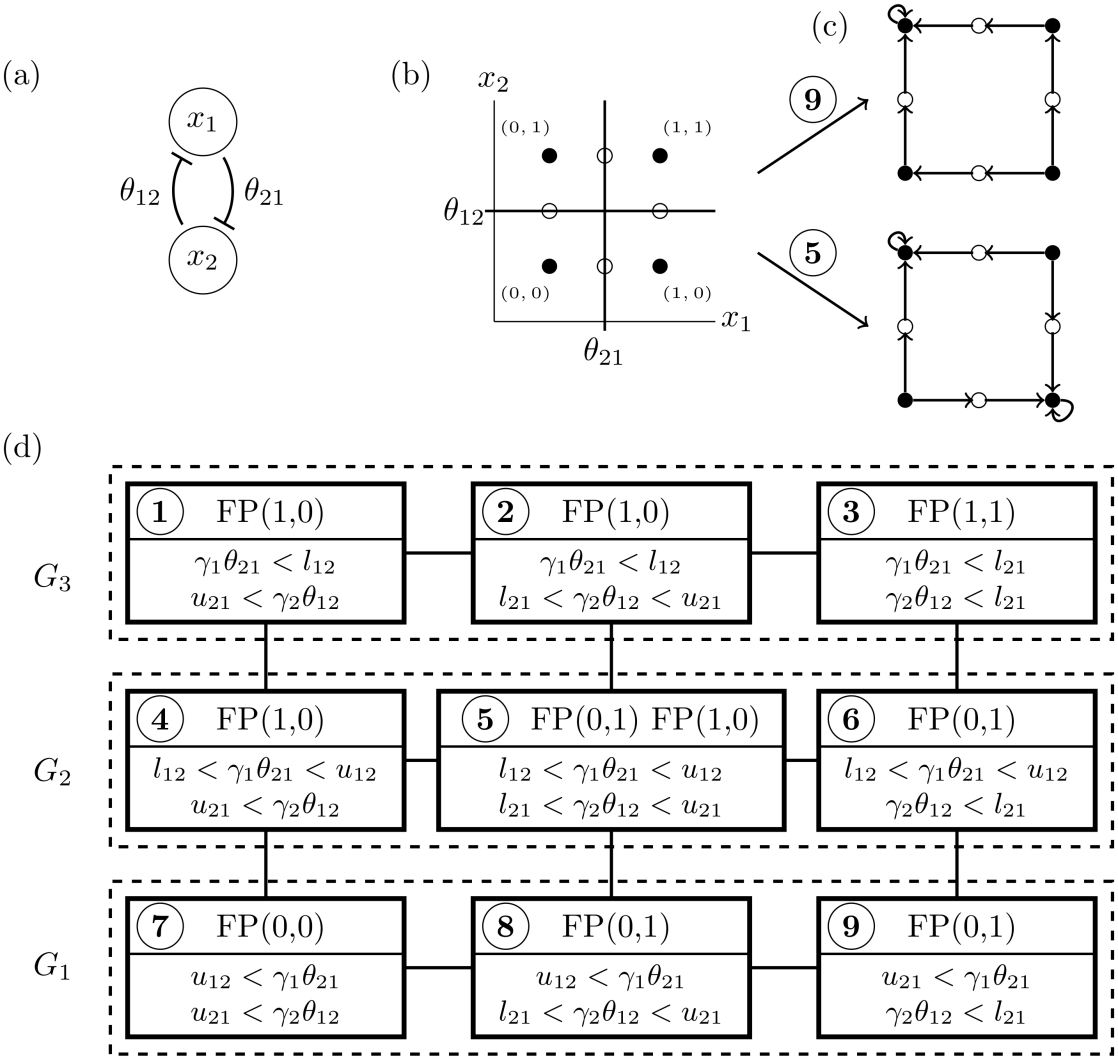
DSGRN for toggle switch. (a) Regulatory network for the toggle switch. (b) Thresholds {*θ*_2,1_, *θ*_1,2_} divide phase space, (0, ∞)^2^, into four 2-dimensional domains (black dots) and 1-dimensional walls separating the domains (circles). (c) For each choice of parameters, given a domain there is a well-defined direction of dynamics at walls that defines the state transition graph (see Fig 6 for the derivation of this dynamics). State transition graph for parameters in regions **5** and **9** of the parameter graph. The Morse graph for **9** consists of a single node FP(0, 1) where FP indicates that the node is terminal in the Morse graph (a trivial statement for this simple example) and that the vector representation of the associated region is (0, 1). The Morse graph for **5** contains two nodes FP(0, 1) and FP(1, 0). (d) Visual interpretation of SQL DSRGN database for toggle switch organized as a parameter graph with explicit description of parameter domains (inequalities in bottom of each square) and Morse graphs (top part of each square) valid over corresponding parameter domain. Dashed boxes indicate elements of *PG*(¬1) = {*G*_1_, *G*_2_, *G*_3_}.

While this simple model of a bistable switch provides intuition for the analysis performed in this paper, experimental data leads us to entertain the possibility that the dynamics of switches in biological systems may be more complex. For example, the lac operon is among the most carefully studied regulatory networks that exhibits bistability. Associated experimental data [20, Fig 2b] leads to a blurred version (with measurement on the vertical axis presented using a logarithmic scale) of the simple single valued curve of Fig 1. With this in mind, the hysteresis phenomenon detected by DSGRN consists of identifying well defined regions that contain the attractors associated with Off and On states. As is made clear in the Materials and Methods section, whether these attractors are stable fixed points or not depends on details of the particular differential equation used in the model.

A network that may be responsible for the restriction point dynamics in mammalian cells was suggested by [21] and then further elaborated by Yao et al. [4]. The essential elements of the restriction point network is a family of E2F transcription factors which are sequestered in a heterodimer by Rb in non-proliferating cells in G1 phase. Release of E2F by phosphorylation of Rb results in initiation of S phase of the cell cycle.

The principal controls of Rb are cyclin/kinase complexes CycD/Cdk4,6 and CycE/Cdk2. CycD/Cdk4,6 is up-regulated by Myc which responds to the cell growth; the initial phosphorylation of Rb by CycD/Cdk4,6 releases E2F, which up-regulates the second kinase CycE/Cdk2, which then completes the phosphorylation of Rb and finishes the release of E2F [4, 17–19, 21]. Since one of the hallmarks of cancer is sustained proliferation in cells that are immune to external signals that would prevent proliferation in normal cells, it is not surprising that dysregulation of this network is observed in the majority of cancers [22]. For recent comprehensive reviews on the connection between retinoblastoma protein (Rb), a key member of this network, and cancer, see [23–26]).

This system exhibits resettable bistability [4] if, as the growth factor input is reduced to zero, bistability vanishes and the cell returns to a non-proliferating phenotype with E2F sequestered, e.g. in Fig 1 as λ is reduced the system moves from bistability to the monostable state Off.

Observe that resettable bistability, and hysteresis are physiological phenomena that can only be expressed via an understanding of global dynamics over paths in parameter space.

Yao *et. al.* [4] executed a modeling study of the mammalian cell cycle restriction point with the goal of identifying “the basic gene circuit underlying resettable Rb-E2F bistable switch by the criterion of robustness” where robustness is defined in terms of the ability to maintain functionality against perturbations. Note that even an idealized description of the restriction point network has multiple variables and a multitude of parameters. Thus, from the mathematical perspective to rigorously carry out the program proposed in [4] requires mathematical and efficient computational techniques capable of addressing at least three fundamental challenges:

1. identify the existence of all parameters exhibiting bistability with minimal knowledge of the functional form of nonlinearities;
2. identify the existence of curves in parameter space over which resettable bistability or hysteresis take place; and
3. provide a systematic quantification of the extent to which a particular regulatory network is capable of exhibiting these features.

The aim of this paper is to demonstrate that DSGRN is capable of meeting these challenges for moderate sized networks. As is discussed in detail below, DSGRN provides information about the global dynamics for all parameter values, and to the best of our knowledge, is unique in these capabilities.

There are similarities between DSGRN and a variety of other approaches. To the best of our knowledge the novel aspects of DSGRN are that we (1) approximate continuous system by a discrete system (the state transition graph) and (2) via our computations we obtain knowledge about the global dynamics for **all** parameters associated with the model.

Other approaches, for instance CPSS (Continuous parameter space search) [27] chose a particular nonlinearity which in turn determines the parameter space. A query based on existence of stable equilibria is established, regions of parameter space are non-uniformly sampled, and the differential equation is integrated at the chosen parameter values to identify existence or lack of existence of equilibria.

In contrast DSGRN searches for attracting regions (a more robust concept than stable equilibria) and thus the set of nonlinearities for which the computations are provably valid is much larger [7]. Furthermore, there is no sampling of parameter space, instead the dynamics is reported for all possible parameter values.

## Results

### Dynamic Signatures Generated by Regulatory Networks (DSGRN)

We view DSGRN as an algorithm; the input is a regulatory network and the output is a queryable database of the global dynamics for all parameters. This algorithm is based on four essential concepts:

- a *Regulatory Network*, the input;
- the *State Transition Graph*, a finite representation of the dynamics;
- the *Morse Graph*, a compact queriable representation of the dynamics at a given parameter value; and
- the *Parameter Graph*, an explicit finite representation of parameter space.

The DSGRN database consists of the parameter graph along with an association of a valid Morse graph to each node of the parameter graph.

Before turning to the Rb-E2F network we use the toggle switch, which consists of two constitutively expressed repressors that repress each other, in an attempt to focus on the general philosophy and novel concepts associated with DSGRN. For more detailed descriptions see the Methods section and [8]. The regulatory network of the toggle switch has the form of Fig 2(a).

DSGRN requires that a regulatory network be a network for which each node has at least one outgoing edge and there is at most one edge from one node to another node.

To each node *n* in regulatory network, DSGRN associates a real value, e.g., concentration, *x*_*n*_ ≥ 0 and a parameter *γ*_*n*_ > 0 representing the rate of degradation of *x*_*n*_. To each edge *m* → *n* (denoting activation) or *m* ⊣ *n* (denoting repression) in a regulatory network, DSGRN assigns three parameters *ℓ*_*n*,*m*_, *u*_*n*,*m*_ and *θ*_*n*,*m*_ where *ℓ*_*n*,*m*_ and *u*_*n*,*m*_ represent low and high levels of growth of *x*_*n*_, respectively, (in particular *ℓ*_*n*,*m*_ < *u*_*n*,*m*_) that are determined by the value of *x*_*m*_ relative to the threshold *θ*_*n*,*m*_. Observe that for a regulatory network with *N* nodes and *E* edges the dimension of the space of parameters is *D* = *N* + 3*E* and is a subset of [0, ∞)^*D*^.

To construct a state transition graph DSGRN uses the thresholds *θ*_*n*,*m*_ to decompose the phase space into rectangular regions (see Fig 2(b)). DSGRN assigns a vertex to each region (solid dot) and to each face between regions (circle). Furthermore, each solid dot is labeled as a vector where the *n*-th entry indicates the number of thresholds *θ*_*m*,*n*_ with values less than *x*_*n*_ for any *x*_*n*_ in the associated region. For each fixed set of parameter values the state transition graph represents the dynamics of the regulatory network via a directed graph based on the above mentioned vertices. As is shown in Fig 2(c) the edges go from circles to solid dots, from solid dots to circles, and potentially there are self edges on solid dots. Fig 2(c) shows that the edges in a state transition graph are parameter dependent.

Since the size of the state transition graph grows rapidly with the size of the regulatory network, DSGRN stores a minimal representation of the global dynamics using a Morse graph, which is an acyclic directed graph (see Fig 2(d)). Morse graphs are capable of encoding potentially complicated dynamics (see [16]), however, since the focus of this paper is on robust switch behavior, we restrict our discussion accordingly. In the state transition graph the most natural representative of a stable fixed point is a vertex with a unique out edge that is a self edge. In the associated Morse graph this is a terminal vertex labeled FP. Furthermore, since only solid dots have self edges, we identify each such terminal vertex by the vector label of the associated solid dot.

The directed edges in the state transition graph are determined by affine multilinear inequalities involving the parameters. These inequalities are defined at each node in the regulatory network RN according to the out edges, in edges, and the logic governing the interaction of in edges. For each node *n* in the regulatory network the inequalities are organized via a node-graph *PG*(*n*) where edges indicate change of a single inequality. For the toggle switch the node-graphs and their associated inequalities are

PG(1):u12<γ1θ21−l12<γ1θ21<u12−γ1θ21<l12,PG(2):u21<γ1θ12−l21<γ1θ12<u21−γ2θ12<l21.

A node in *PG*(*n*) is called *low* (*high*) if all the associated *ℓ* (*u*) values are less (greater) than all the associated *γθ* values. The remaining nodes are called *intermediate* nodes.

The parameter graph for a regulatory network is the product of the node-graphs, i.e. $PG=\prod_{n=1}^{N}PG\left(n\right)$. A graphical representation of the parameter graph of the toggle switch is given in Fig 2(d). Observe that the inequalities associated with each node provides a subdivision of parameter space. For the toggle switch the parameter graph provides a representation of parameter space, which is an unbounded region of (0, ∞)^8^, via nine regions. Furthermore, it is guaranteed that for every parameter in a given region the associated state transition graph is the same, and therefore, the Morse graph is constant over each region.

Because the parameter graph is a product graph, if we fix a node *n* in the regulatory network, then we can decompose the parameter graph into a collection of subgraphs each one of which is isomorphic to *PG*(*n*). We denote this collection of subgraphs by *PG*(¬*n*). As shown in Fig 2(d), for the toggle switch, *PG*(¬1) = {*G*_1_, *G*_2_, *G*_3_}. The fact that any *G* ∈ *PG*(¬*n*) is isomorphic to *PG*(*n*) implies that a path in *PG*(*n*) defines a path in *G*. We call the path in *G* the *lift* of the path in *PG*(*n*).

We now describe implemented queries to the DSGRN database that are relevant to the analysis of switching behavior. A Morse graph exhibits *(p, q) bistability* if it contains terminal nodes FP = *p* and FP = *q*. Fix a node *n* in the regulatory network and let *G* be a element of *PG*(¬*n*). The subgraph *G* exhibits:

- *(p, q) bistability* if there is a vertex in *G* such that the associated Morse graph exhibits (*p*, *q*) bistability.
- *resettable (p, q) bistability to p* if there is a path in *PG*(*n*) from a low (or high) node to an intermediary node such that for the lifted path in *G*, the Morse graph associated to the lift of the low (high) node has unique terminal node FP = *p*, the Morse graph associated to the lift of the intermediate node exhibits (*p*, *q*) bistability, and the Morse graphs associated with the remaining vertices along the lifted path exhibit either a unique terminal node FP = *p* or (*p*, *) bistability, where * stands for arbitrary FP.
- *hysteresis between p and q* if there exists a path in *PG*(*n*) from low (high) to high (low) nodes that exhibits both resettable (*p*, *q*) bistability to *p* and resettable (*p*, *q*) bistability to *q*.

We demonstrate the biological relevance of DSGRN on the synthetic toggle switch implemented in Gardner *et. al.* [28] using Lac repressor *lacI* and a temperature sensitive phage λ repressor (*cIts*), with externally supplied IPTG as a control. Since IPTG binds directly to the Lac repressor and inactivates it, IPTG effectively lowers the available concentration of the Lac repressor.

Let *x*_1_ represent the concentration of *cIts* and *x*_2_ represent the concentration of *lacI*. The state where *cIts* (*x*_1_) is fully expressed and *lacI* (*x*_2_) is repressed is designated as an ON state, and the state where *x*_1_ is low and *x*_2_ is high is designated as an OFF state. In the DSGRN database (Fig 2(d)) FP(1, 0) and FP(0, 1) correspond to ON and OFF, respectively. Increase in the concentration of IPTG decreases the values of *ℓ*_12_ and *u*_12_ and thus is quantified by a path in *PG*(1) from the high node to the low node.

Visual inspection of Fig 2(d), shows that *G*_2_ is the only subgraph in *PG*(¬1) that exhibits (ON, OFF) bistability. Furthermore, moving monotonically along the path defined by *G*_2_ from node **4** to node **6** results in hysteresis between ON and OFF states. Thus the DSGRN database analysis suggests that if the toggle switch is operating at the bistable regime (node **5**), then sufficiently strong IPTG treatment phenotype leads to FP(1, 0), which represents the ON state. This agrees with the experimental observations [28].

In the parameter graph there are parameter nodes, called *inessential parameter nodes*, for which at the associated parameter values there is a node *n* in the regulatory network such that the inequalities that determine the state transition graph do not vary as a function of *x*_*n*_. At inessential parameter nodes the network dynamics is identical to that of a subnetwork of the regulatory network. In the computations presented in the remainder of the paper we only consider *essential subgraphs* *EPG*(¬*n*) ⊂ *PG*(¬*n*), i.e. subgraphs of the parameter graph that do not contain any inessential parameter nodes.

### Restriction point in mammalian cell cycle

The E2F-Rb network regulates the restriction point of the mammalian cell cycle, i.e. the point where the progression through the cell cycle decouples from the growth signals [4, 17–19]. The E2F-Rb network exhibits two essential phenotypes: when the growth signal is absent, the transcription factor E2F is sequestered in the heterodimer with Rb. This is the *quiescent state* (QS). On the other hand, when the growth signal is present at high level, E2F disassociates from the E2F-Rb dimer and activates numerous downstream processes. This *proliferative state* (PS) initiates entry into *S*-phase of the cell cycle.

**Fig 3:**
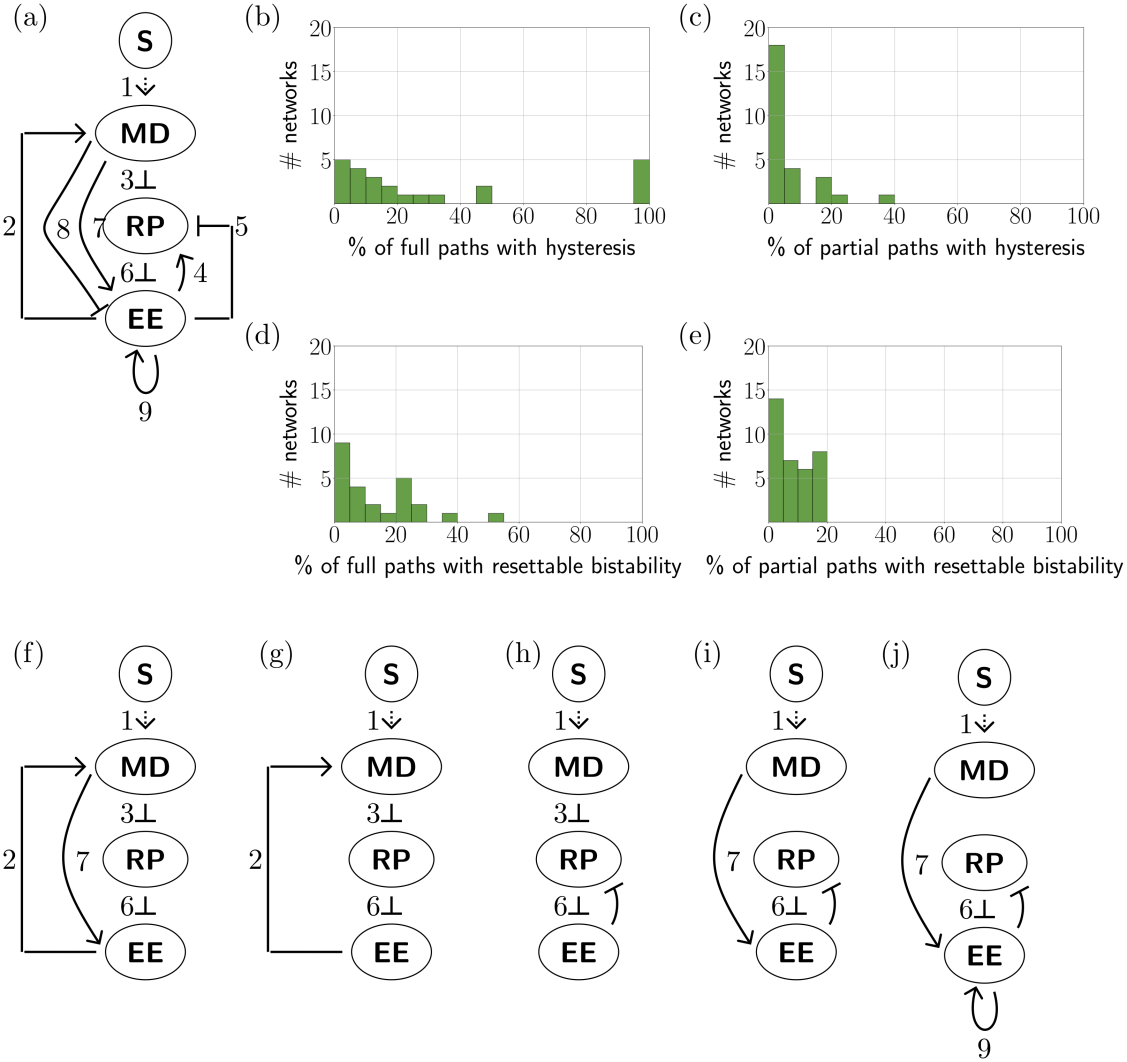
3 node E2F-Rb networks. (a) 3 node network with potential edges from [4] with signal *S* acting on *MD*. (b) Histogram showing 24 regulatory networks expressing full path hysteresis between QS and PS. Five regulatory networks show full path hysteresis in 100% of *EPG*(¬*MD*). (c) Histogram showing 27 regulatory networks expressing partial path hysteresis between QS and PS. (d) Histogram showing 25 regulatory networks expressing full path resettable (QS,PS) bistability to QS. (e) Histogram showing 35 regulatory networks expressing full path resettable (QS,PS) bistability to QS. (f)-(j) Five networks that exhibit full path hysteresis along all appropriate paths in *EPG*(¬*MD*) These networks are also the top five networks in partial path hysteresis in descending order (f),(g)-(i), (j). Network (f) is also highest in prevalence of partial path resettable bistability, but does not show any full path resettable bistability. Full results for all networks can be find in S1 Table, where the five networks (f)-(j) have numbers 24,39,46,26 and 22.

Yao *et. al.* [4] executed a modeling study of the mammalian cell cycle restriction point with the goal of identifying “the basic gene circuit underlying resettable Rb-E2F bistable switch by the criterion of robustness” where robustness is defined in terms of the ability to maintain functionality against perturbations. In particular, they start with a large network from [17, 18] that, as is indicated in Fig 3(a), they coarse-grain into a system with three nodes and a variety of possible edges. By considering connected subnetworks of Fig 3(a) they construct a library of 768 mathematical models of networks. They assume that interactions between the nodes are governed by Hill-functions, thus producing a model consisting of a three dimensional system of ordinary differential equation model with up to 26 parameters. To evaluate the models they generate 20,000 parameter sets by randomly sampling from reasonable parameter ranges for each of the 26 parameters. Each of the 768 networks is given a score based on the percentage of this collection of parameters at which the differential equations exhibits a particular switching characteristic like resettable bistability or hysteresis.

There are several mathematical objections that can be made to this procedure. First, if one were to generate the random parameters by insisting on at least two independent choices for each parameter, then one would need to consider 2^26^ ≈ 7 × 10^7^ parameter sets. It is insufficient to sample dynamics at distinct parameter values as a proxy for its prevalence without a priori bounds on the sensitivity of this dynamics to changes in the parameters. Second, it is not clear how well a Hill function approximates the nonlinear behavior of the system. Third, as indicated in the introduction, the phenomena of resettable bistability and hysteresis are both a function of a continuous change of parameters. Therefore it is insufficient to sample dynamics at distinct values of the input variable *S* as a proxy for presence of these phenomena.

We now show that DSGRN can efficiently replicate the efforts of [4]. We begin with a discussion of how it avoids the above mentioned mathematical concerns. First, DSGRN allows one to identify any point in parameter space with a node in the associated parameter graph; in turn, each node in the parameter graph is identified with a region of parameter space for which the dynamics can be described via a Morse graph. Therefore, with DSGRN one does not restrict the analysis to finite collections of parameters; the analysis is valid for all parameter values. Second, the DSGRN analysis is based on representing dynamics via state transition graphs and, for the purposes of this paper, interpreting the dynamics via terminal nodes in the Morse graph. The terminal nodes represent regions in phase space that are trapping regions for broad classes of nonlinearities [7]. Hence, with DSGRN one is not restricted to a specific analytic representation of the dynamics. Third, as is demonstrated in the toggle switch example, paths through the parameter graph represent continuous paths through parameter space, thus questions of resettable bistability or hysteresis can be rigorously addressed. In addition, because there are finitely many lifts of parameter paths in the parameter graph we can quantify the number of lifts for which the desired switching phenomenon does occur.

There are 49 regulatory subnetworks of Fig 3(a), such that every node has at least one out-edge, and there is no more than one edge from one node to another. For each of these subnetworks we compute *EPG*(¬*MD*), where node *MD* is singled out since the input signal *S* impacts the network at the node *MD*. Varying the input of the network corresponds to a path in *EPG*(¬*MD*). We assume that a monotone change in the strength of the signal *S* acts monotonically on *MD*, but we do not necessarily assume that we know the range of *S*. This leads us to consider two cases.

In the first case we assume that the network parameters are “aligned” with the range of the signal. In this case as *S* ranges from its lowest value to its highest value, *MD* moves from lowest quantifiable level, i.e. being below all the thresholds associated with *MD*, to the highest quantifiable level, i.e. being above all the thresholds associated with *MD*. In terms of paths in *EPG*(¬*MD*), this is associated with a *full path*, that is, any path that starts at the parameter node where all outputs of the node *MD* are below all thresholds of the nodes *MD* connects to, and finishes at the parameter node where all outputs of the node *MD* are above all thresholds of the nodes *MD* connects to.

In the second case we do not assume that the range of the signal *S* is matched to range of *MD*. In terms of paths in *EPG*(¬*MD*) this is modeled by a *partial path*, i.e. any subpath of the full paths. Note that the extreme case of a partial path is a constant path, that is, a path consisting of a single node in *EPG*(¬*MD*). Physically, this implies that the variance of the input signal is not sufficiently large to impact the dynamics of the regulatory network.

We remark that given a regulatory network it is straightforward to determine the collection of monotone full and partial paths of *EPG*(¬*MD*); however, the total number of paths is network dependent.

It is important to note that in order to exhibit resettable bistability (hysteresis) the parameter graph *EPG*(¬*MD*) must contain monotone paths with at least two (three) nodes, respectively. This, in turn, implies that the parameter graph for *MD* must contain at least two (three) nodes. Therefore networks where *MD* has a single outgoing and no incoming edge cannot exhibit hysteresis under our approach. We view this as a technical failing of DSGRN induced by our insistence on only using coarse measurements defined in terms of the thresholds. To circumvent this problem, we note that the assumption that in the regulatory network *MD* has a single outgoing and no incoming edge implies that while *MD* talks to the network, it is not affected by the network. In particular, returning to our assumption that *MD* behaves monotonically with respect to the signal *S*, this implies that we can without loss of generality assume that the signal acts in a monotone fashion on the unique node that *MD* acts on. Of course, whether we assume that the signal acts on this node in a monotone increasing or decreasing manner depends on whether the out edge from *MD* represents activation or inhibition.

For the regulatory subnetworks of Fig 3 we order the nodes by (MD, RP, EE). We interpret the quiescent and proliferative states of the restriction point network in terms of *dynamic signatures* of DSGRN. In the quiescent state QS, E2F is sequestered in the heterodimer with Rb and therefore the levels of free E2F are low. In the 3-node network in Fig 3(a) free E2F is represented as EE. Following [4] we associate the quiescent state with low levels of EE and the proliferative state with high levels of EE. In the DSGRN database the precision to which we can search for attractors is limited by the number of thresholds. In other words, we can identify if coordinates associate with an attractor are bounded between consecutive threshold values of that variable, but any finer identification requires choice of a particular nonlinear differential equation and a choice of particular parameter values. The number of thresholds of a variable is determined by the number of edges emanating from a node that corresponds to the variable, since each such edge is associated to a single distinct threshold. We characterize quiescent state QS as a minimal mode in the Morse graph with labeling FP(*, *, 0).

Similarly, the proliferative state PS is characterized by high levels of free E2F (represented by EE), and therefore a PS phenotype will be represented by an attractor that has the EE coordinate above at least one threshold. Since the number of thresholds of EE changes depending on the subnetwork that we analyze we characterize this attractor as a minimal mode in the Morse graph with labeling FP(*, *, *m*), for some *m* ≥ 1.

We are now in a position to compute statistics that measure the robustness of the subnetworks with regard to the phenotypic behaviors of resettable bistability and hysteresis. In particular given a subnetwork, we define its *prevalence of full path resettable bistability* and *prevalence of full path hysteresis* to be the number of full paths in *EPG*(¬*MD*) that exhibit resettable (QS, PS) bistability to QS and hysteresis normalized by the number of full paths in *EPG*(¬*MD*), respectively. Similarly, we define *prevalence of partial path resettable bistability* and *prevalence of partial path hysteresis* to be the number of partial paths in *EPG*(¬*MD*) that exhibit resettable (QS, PS) bistability to QS and hysteresis normalized by the number of partial paths in *EPG*(¬*MD*), respectively. Note that although a full path is also a partial path, the different normalizations do not allow one to make a priori conclusions on the relative values of full path prevalence and partial path prevalence for either phenotype.

These numbers provide different information about the networks. As indicated above, prevalence based on full paths assesses the ability to achieve a given phenotype when the input range matches the range of *MD*, while prevalence based on partial paths provides information about the behavior of the network where no assumption is made about these ranges.

Fig 3(b)–3(e) provides histograms indicating the number of subnetworks of Fig 3(a) for which there is positive prevalence of full and partial path hysteresis and resettable bistability. Focussing on the full path hysteresis, there are five networks (Fig 3(f)–3(j)) that exhibit hysteresis for every full path in *EPG*(¬*MD*).

More generally, the networks (Fig 3(f)–3(j)) are also the top five networks with respect to prevelance of partial path hysteresis. Network in Fig 3(f) shows partial path hysteresis in 20% of paths, networks Fig 3(g)–3(i) in 16.66%, and Fig 3(j) in 15.49% of all the partial paths. Furthermore, all the networks Fig 3(f)–3(j) are among the top 8 networks that exhibit the greatest prevalence for partial path resettability. Interestingly, none of these networks rank among the top 8 networks for full path resettable bistability and one of them, network in Fig 3(h), has no full path with resettable bistability. The best two subnetworks found in [4] appear among the top 8 in full path resettable bistability and among top 10 in full path hysteresis, but not among the top eight in either partial path hysteresis or resettable bistabilty.

It is worth contemplating why the results using DSGRN do not agree exactly with the results of [4] (aside from the obvious fact that we only consider networks that involve all three nodes) and the biological significance of these differences. There are at least three fundamental differences in the approaches.

1. As indicated earlier the results of [4] are based on sampling of parameter space via 20,000 sets of parameter values, whereas DSGRN is computed over all associated parameter values. Thus, it is not surprising that there is not complete agreement with respect to the orderings. What is not clear is which result is more biologically relevant for this particular problem. It is easy to imagine that [4] has under-sampled parameter space. However, since DSGRN examines all of parameter space, it also possible that our count includes parameter values that are not biologically relevant. However, this concern is also applicable to the work of [4].
2. The bistability measurement of [4] is a measurement of particular parameter values at which bistability occurs. Similarly, resettable bistability is a measure of pairs of sets parameter values. DSGRN identifies paths through parameter space such that along the path there exist appropriately ordered sets of parameter values at which bistability and monostability occur. If one assumes that the signal acting on the systems takes on continuous values, then DSGRN provides a more appropriate model on which to count.
3. In [4] the dynamics is modeled using Hill function nonlinearities. One of the parameters in this model is the value of the exponent. Since [4] chooses the parameter values at random, some fraction of the sampled systems involve relatively low values of the exponent, which may be a significant feature of the biochemical reactions. DSGRN is based on switching functions and thus from a functional perspective is close to Hill functions with high values of the exponent, and in this sense, DSGRN is sampling from a more restrictive subset of parameter values. At the same time since DSGRN does not depend on the precise form of the Hill function, it captures the dynamics of a wider variety of nonlinearities.

### 5 node restriction point networks

**Fig 4:**
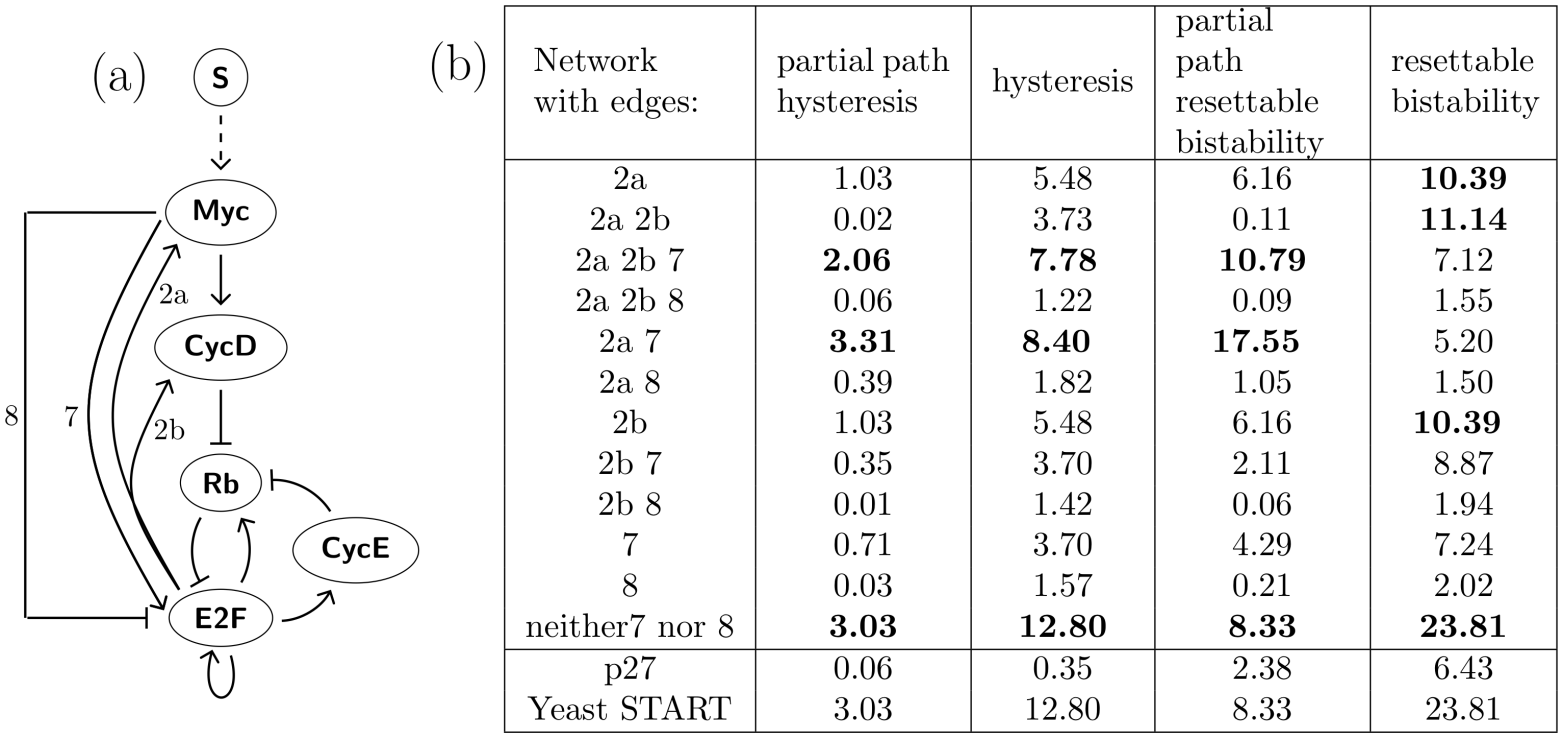
Search for the best 5 node E2F-Rb network. (a) Full 5 node E2F-Rb network with input *S*. We test 12 subnetworks, listed in Table (b), top, for robustness of partial path and full path resettable bistability and hysteresis. (b) Each of the 12 subnetworks contains the unmarked edges in (a). In addition, we either add one of the edges 7 or 8, and/or a subset of the pair of edges (2a, 2b). The second through fifth columns list the percentage of subgraphs in *EPG*(¬*MD*) satisfying indicated query. Top three results in each column are emphasized. Note that top three networks in first three queries agree. While the very best network under full path resettable bistability is the same as for full path hyesteresis, none of the top three networks for full path hysteresis has either edge 7 or the edge 8.

The 3-node networks of the previous section were derived as simplification of [4, Fig 1(A)]. With this in mind we return to [4, Fig 1(A)] and consider less radical simplifications that result in the 5-node networks indicated in Fig 4(a). In particular, we replace the MD node by two nodes Myc and CycD representing cyclin/kinase complex CycD/Cdk4,6, with the assumption that Myc up-regulates CycD, and the EE node by E2F and CycE, representing cyclin/kinase complex CycE/Cdk2, with the assumption that E2F up regulates CycE. The node Rb represents free and active form of Rb proteins. We also include all the potential edges from Fig 3(a) with appropriate modifications of beginning and ending nodes. We repeat the bistability, resettable bistability and hysteresis queries from the previous section on *EPG*(¬*MD*) for the regulatory network subnetworks indicated in Fig 4(b).

The unlabeled edges are included in all computations with additional edges listed in first column of table in Fig 4(b). We organize the networks into three groups: networks that have edge 7, networks that have edge 8, and networks that have neither.

The prevalence of partial path and full path hysteresis, as well as partial path and full path resettable bistability is indicated in the columns of Fig 4(b). In each column we highlight top three or four values. Note that the best three networks are the same under the measures of partial and full path hysteresis and partial path resettable bistability. The network that is in top three in every category is network that does not have either edge 7, nor 8 and does not have either 2a, nor 2b.

On the other hand, full path resettable bistability orders networks differently. The top network is still the same. Existence of edges 7 and 8 is still undesirable, but a network with the edge 2a, a network with edge 2b alone, and some networks with both 2a and 2b rank very highly.

The subtle difference between partial path and full path resettable bistability stems from the fact that full path resettable bistability requires that there are only two Morse graph types along the entire path: either the bistable state and the terminal state to which the bistable state resets. In the context of E2F-Rb network this corresponds to a set of states (QS, …, QS, …, B,…, B) where *B* is a bistable state (*QS*, *PS*). A full path hysteresis does not correspond to a full path resettable bistability, since there are different states at the ends of the path. In E2F-Rb networks the full hysteresis corresponds to states (QS, …, QS, …, B, …,B, …,PS, …PS). However, a full path hysteresis gives rise to a partial path resettable bistability by considering only first half of the full path.

**Fig 5:**
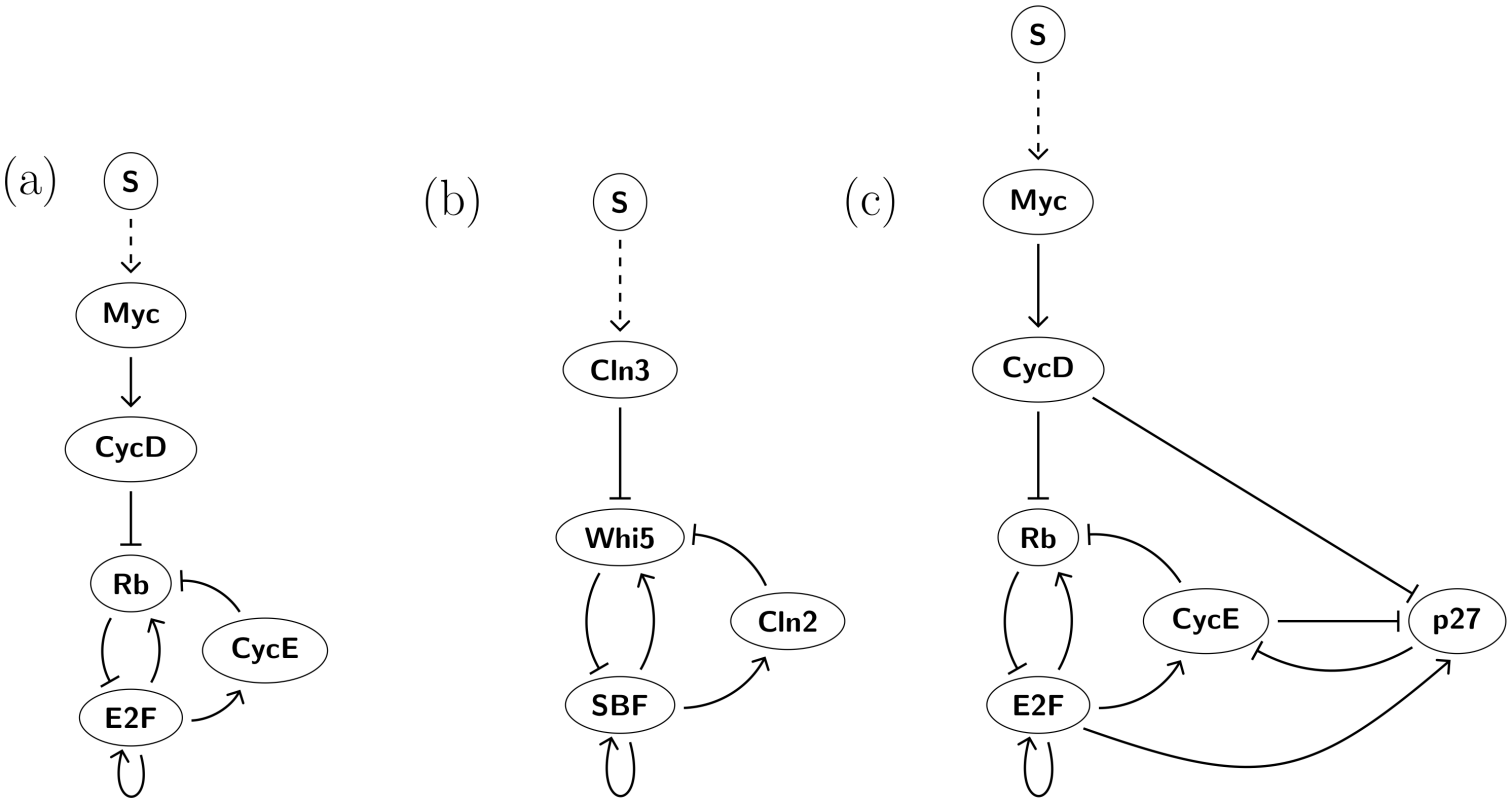
Comparison between human and yeast networks. (a) The best 5-node network from Fig 4(b) that exhibits the most robust full path and partial path hysteresis. (b) Cell cycle initiation network from yeast (START network) [29, 30] exhibits robust resettable bistability in 23.81% of the full paths 12.8%. Because the networks in (a) and (b) only differ by a node with a single input and single output (*Myc*), the networks in (a) and (b) will give the same results in our analysis. (c) Best 5-node network from Fig 4(b) with added p27 shows full path resettable bistability in 6.43% and full path hysteresis in 0.35% of the corresponding parameter paths.

For comparison, we study the yeast cell cycle initiation network (START), see Fig 5(b) [29, 30]. The START network of the budding yeast cell cycle has the same topology as E2F-Rb networks, yet there is no homology among the protein and transcription factors in the two networks [29, 31]. A transcription factor SBF is sequestered by Whi5 during G1. The cell growth leads to accumulation of cyclin/kinase complex Cln3/Cdk1 which phosphorylates Whi5 and as a result, releases SBF from the complex. Released SBF promotes expression of another cyclin Cln2, which is part of a cyclin/kinase complex Cln2/Cdk1.

This complex in turn finishes phosphorylation of Whi5 and completes the release of SBF [30, 32, 33]. The analogy with the mammalian restriction point network in Fig 4(a) is striking. The results for the START network are in the table in Fig 4(b). We note that the best of the 12 subnetworks of mammalian E2F-Rb network is the network that does not have edge 7,8, 2a or 2b. We assume that in this network S acts monotonically on Myc, which in turn acts monotonically on CycD, which acts monotonically on Rb. Thus, as is described above, our analysis of this network reduces to the analysis of the 3 node network involving only the nodes Rb, CycE, and E2F with repressive input on Rb. This reduced 3 node network is identical to the similarly reduced yeast START network, therefore the numbers in the corresponding rows in table in Fig 4(b) are identical.

We conclude that the START network matches the subnetwork of the mammalian E2F-Rb network, that most robustly exhibits both full path resettable bistability and hysteresis. The similarity of E2F-Rb network and yeast START network led to a hypothesis that what is maintained in the process of evolution is network structure and function [31]. Our results support this hypothesis, since the core subnetwork exhibiting most robustly the expected phenotype of a switch is common between E2F-RB mammalian and yeast START networks.

### Restriction point network with p27

Starting with the best 5-node network, we add effects of kinase inhibitor p27, which prevents activation of cyclinE/Cdk2 complex (CycE) (see Fig 5(c)). p27 is sequestered by the cyclinD/Cdk2,4 (CycD) complex, as well as tagged for ubiquitination by CycE. Increased levels of p27 protein typically result in arrest in G1 [34].

Using the regulatory network of Fig 5(c) we measure prevalence of (QS, PS) resettable bistabilty and hysteresis in *EPG*(¬*Myc*). The p27 network still shows resettable bistability and hysteresis, see Fig 4(b), but at the levels below the levels observed in the best 5 node network Fig 5(a) and the yeast START network Fig 5(b). Since p27 is a part of other cellular control mechanisms responsible for initiation of cellular arrest, and we have not included these in our network, there is no reason to believe that addition of p27 should make the switching phenotype of the E2F-Rb more robust. Therefore we do not expect that the prevalence of hysteresis and resettable bistability in p27 network will be higher than in the the best 5 node network in Fig 5(a). Being part of of a control network that provides ability to arrest cell cycle, p27 provides capabilities that are not captured by the assessment of the robustness of the switch.

We also note, that when we tested this network for (QS,PS), rather than the (PS,QS), hysteresis and resettable bistability, we found there are no parameter paths that exhibit either hysteresis, or resettable bistability, either partial or full path. This suggests that, while not as robust as in its subnetworks, the phenotype of the switch from QS to PS is an important aspect of the network design.

## Discussion

We present a new modeling and computational platform that can rapidly compute rigorous summaries of dynamics over large regions of parameter space. The description of the dynamics is fine enough to distinguish attractors with different expression levels, find bistability, resettable bistability and hysteresis; while not the focus of this paper, DSGRN can also find oscillatory behavior [9].

We validate our approach using the toggle switch and the E2F-Rb network responsible for mammalian restriction point dynamics. In particular, in contrast to the approach adopted by Yao [4] to investigate the the prevalence of resettable bistability in the parameter space of 3 node networks based on Hill type models by running simulations of the Hill type models at 20,000 fixed parameters, we use DSGRN to search for resettable bistability and hysteresis over the entire parameter space.

We quantify the prevalence of these two phenotypes by counting paths connecting regions of the parameter space along which dynamics shifts from quiescent state to bistability (for resettable bistability phenotype), or from quiescent state through bistability to proliferative state (for hysteresis phenotype). These paths represent the response of the network to an external input *S*. Since we do not necessarily assume that the network parameters are aligned with the range of the signal *S*, we count both full paths, that represent a full range of the network response and partial paths, that are subpaths of the full paths.

The computational efficacy of DSGRN allows us to perform the same calculations on several 5-node networks that include explicit representations of CycD/Cdk4,6 and CycE/Cdk2, and on a 4 node START network in the yeast that is functionally and structurally similar to the E2F-Rb network, as well as 6-node network which includes CycD/Cdk4,6, CycE/Cdk2 and p27. We are not aware of any other approach which computes a description of the global dynamics of a 6-dimensional system over the entirety of a 39-dimensional parameter space.

Our computations show that out of the twelve 5-node subnetworks of the network Fig 4(a) the one that most robustly shows both full path hysteresis and resettable bistability does not have edges 2a,2b, 7 or 8. This is the network that is, apart of the presence of *Myc*, identical to the START network in yeast. Both show full path hysteresis in 12.8% of full paths and 23.81% of full paths of the relevant parameter graph. This indicates significant robustness of the switch-like behavior. Wang *et.al.* [29] pose the question: which mechanisms may be responsible for converting a relatively small change in total Cln3 into a large effect in switching on SBF in START network? Super-sensitivity, and cooperativity were mentioned as potential explanations. Our approach allows for the analysis of the the dynamics of networks with 4-7 nodes and the results do not seem to be immediately explainable by properties of its smaller subnetworks with 2 or 3 nodes. The robustness of the observed dynamics may be an emergent property of the entire network.

This conclusion presents a challenge to the current paradigm of motifs, that assumes that we can achieve understanding of complex networks by studying smaller, accessible motifs with few nodes [1]. The hope is that methods like those presented in this paper may be used to directly probe dynamics of larger networks.

## Materials and methods

### DSGRN

We provide a brief description of the steps involved in the DSGRN computations but refer the reader to [8] for complete details.

A regulatory network is a finite directed graph with edges annotated by *j* → *i* or *j* ⊣ *i* (but not both) indicating up and down regulation of *i* by *j*, respectively. We allow vertices to have zero or more in-edges and one or more out-edges.

Each node *i* with more than one incoming edge has an assigned *logic*, expressed in terms of *and* or *or* statements of the inputs, that describes how the information is processed.

The passage from a regulatory network to a state transition graph is dependent upon the parameter values *γ*, *ℓ*, *u* and *θ* described earlier and based on the following constructions. Observe that fixed threshold values {*θ*_*n*, *k*_}, give rise to an explicit decomposition of the phase space [0,∞)^*N*^ into *N*-dimensional cells (see Fig 2(b)). To avoid degenerate cells we assume that for all *j* ≠ *k*

$$\begin{array}{c} \theta_{n,j}\neq \theta_{n,k},\;n=1,\ldots ,N. \end{array}$$ (1)

These are called the *n-threshold inequalities*. Under this assumption the number of *N*-dimensional cells is always the same, independent of the particular choice of parameters. Furthermore, since the boundaries of the *N*-dimensional cells are determined by the thresholds, we can label any cell *κ* by an integer vector *κ* = (*κ*_1_, …, *κ*_*N*_) by setting *κ*_*n*_ to be the number of thresholds *θ*_*j*, *n*_ below the *x*_*n*_ component of an arbitrary point *x* ∈ *κ*. The *vertices* of the state transition graph are the *N*-dimensional cells and their *N* − 1-dimensional faces.

As indicated earlier there are three types of *edges* in the state transition graph: (i) from an *N*-dimensional cell to one of its *N* − 1-dimensional faces, (ii) from an *N* − 1-dimensional face to an *N*-dimensional cell of which it is a face, or (iii) a self edge on an *N*-dimensional cell. Recall that *N*-dimensional cell has a self edge if and only if there it has no edges of type (i). The choice of edges of type (i) and (ii) is determined by the parameter dependent inequalities, called the *n-field inequalities*,

$$\begin{array}{c} 0\neq -\gamma_{n}\theta_{j_{k},n}+\Lambda_{n}\left(\kappa \right),\;k=0,1,\;\text{and}\;n=1,\ldots ,N \end{array}$$ (2)

explained below.

As indicated earlier parameters are assigned to each edge. These parameters are used to define a step function according to the rules:

$$\begin{array}{c} \text{if}\;k\to n\text{,}\;\text{then}\;\sigma_{n,k}^{+}\left(x_{k}\right)=\{\begin{array}{cc} \ell_{n,k} & \text{if}\;x_{k}<\theta_{n,k}\text{,}\\ u_{n,k} & \text{if}\;x_{k}>\theta_{n,k}\text{,} \end{array} \end{array}$$

and

$$\begin{array}{c} \text{if}\;k⊣n\text{,}\;\text{then}\;\sigma_{n,k}^{-}\left(x_{k}\right)=\{\begin{array}{cc} u_{n,k} & \text{if}\;x_{k}<\theta_{n,k}\text{,}\\ \ell_{n,k} & \text{if}\;x_{k}>\theta_{n,k}\text{.} \end{array} \end{array}$$

Since by assumption *ℓ*_*n*, *k*_ < *u*_*n*, *k*_, the implied biological interpretation is that $\sigma_{n,k}^{+}$ ($\sigma_{n,k}^{-}$) models activation (repression) of the *n*-th species by the *k*-th species. For node *n*, a function Λ_*n*_ is defined in terms of sums (if the logic indicates *or*) and products (if the logic indicates *and*) of the step functions $\sigma_{n,k}^{\pm }$. Observe that Λ_*n*_ is constant on any *N*-dimensional cell *κ*. We denote this value by Λ_*n*_(*κ*).

**Fig 6:**
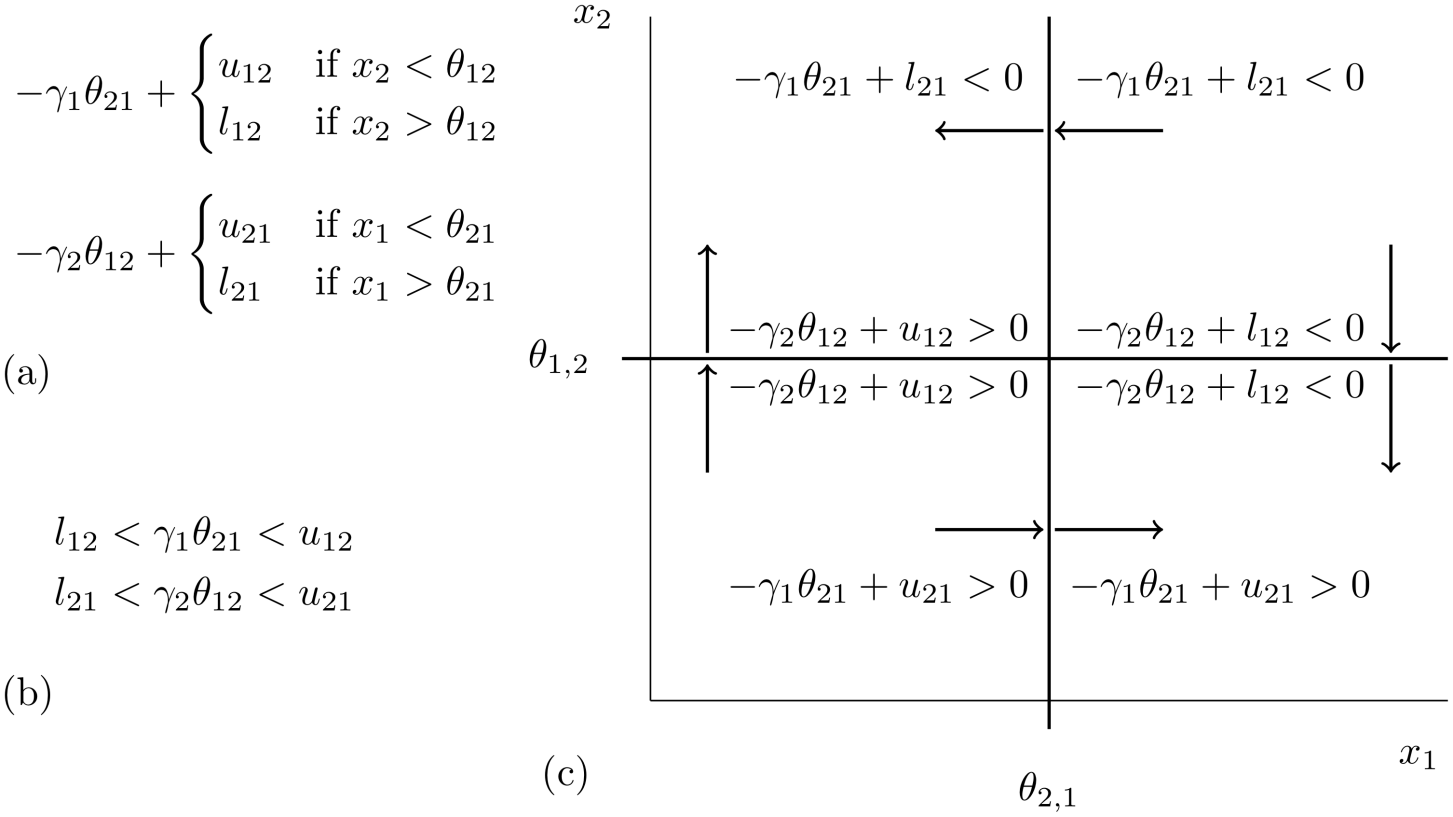
(a) Field equations for toggle switch. (b) Parameter values that are associated with parameter node **5**. (c) Evaluation of field inequalities at four 1-dimensional faces (walls) of domains based on the given parameter value. Arrows indicate direction of dynamics induced by inequalities.

Because of the biological interpretation of the parameter values the quantity *γ*_*n*_*x*_*n*_ + Λ_*n*_(*κ*) indicates the growth rate of species *x*_*n*_. In the *x*_*n*_ direction the *N*-dimensional cell *κ* is typically bounded by two *N* − 1-dimensional faces whose *x*_*n*_ coordinate values are threshold values that we label as *θ*_*j*_*k*_,*n*_, *k* = 0,1. Observe that (2) indicates whether an initial condition on the *N* − 1-dimensional face *θ*_*j*_*k*_,*n*_ flows into or out of the *N*-dimensional cell *κ*. In the former case the state transition graph edge is of type (i) and the latter case the state transition graph edge is of type (ii). Fig 6 indicates the application of these ideas in the context of the toggle switch.

To give proper perspective to our transition from a regulatory network to a state transition graph it is important to recall the notion of a *switching system*

$$\begin{array}{c} {\dot{x}}_{n}=-\gamma_{n}x_{n}+\Lambda_{n}\left(x\right),\;n=1,\ldots ,N, \end{array}$$ (3)

introduced by Glass and Kaufmann [35, 36]. In particular, while it is absolutely clear that switching systems motivate our modeling approach, our results are not limited to this particular system of differential equations. For the analysis performed in this paper we focus on the existence of FP(*κ*) where the *N*-cell *κ* has an edge of type (iii). Consider any system of differential equations $\dot{x}=f\left(x\right)$ where the vector field *f* points in the same direction on the boundary of *κ* as Λ. Under the induced dynamics, there exists a compact attractor in the interior of *κ*. Of course, in the special case that *f*(*x*) = Λ(*x*), i.e. that we have a switching system, then there exists a unique stable fixed point in the interior of *κ*. In summary, the results obtained via DSGRN are not only applicable to switching systems, but to a much large class of nonlinear models (see [7] for a detailed discussion in planar systems).

In the switching system literature the closest to our perspective are papers [37, 38] that emphasize combinatorial aspects of the dynamics of switching systems. However, the emphasis is on analysis of a particular state transition graph, rather than consideration of the entire collection of transition graphs that can be generated by a given network.

The state transition graph grows rapidly with respect to the number of nodes and edges in the regulatory network. Furthermore, as is shown in [39] even switching systems can exhibit complicated dynamics and thus it is not surprising that the dynamics represented by the state transition graph can also be complicated. Thus, to construct a finite queryable representation of the global dynamics requires a coarse description of the dynamics.

To compress this information we construct the associated Morse graph that captures the essential recurrent and nonrecurrent dynamics. A recurrent component in a state transition graph is a maximal subgraph that contains at least one edge and for any two vertices (perhaps the same) there exists a path from one to the other. The recurrent components of the state transition graph make up the vertices of the Morse graph. Since any recurrent component must contain at least one *N*-dimensional cell, we label each vertex in the Morse graph by the collection of labels of all the *N*-dimensional cells in the associated recurrent component of the state transition graph. Observe that there is a natural partial ordering on the vertices of the Morse graph; given two recurrent components *M*_*i*_ and *M*_*j*_ in the state transition graph we set *M*_*i*_ > *M*_*j*_ if there is a path in the state transition graph from *M*_*i*_ to *M*_*j*_.

The focus of this paper is on the existence of FPs, thus we restrict our attention to minimal vertices of the Morse graph derived from recurrent components consisting of a single vertex in the state transition graph. However, in general a vertex in the Morse graph can arise from a recurrent component of the state transition graph that captures complex, even chaotic, dynamics. Thus, the DSGRN is capable of encoding a wide variety of different dynamical structures.

We now turn to a discussion of the parameter graph. A nonempty region defined by particular instantiations of the *n*-threshold inequalities (1) and the *n*-field inequalities (2), e.g. $\theta_{n,j}{}_{<}^{>}\theta_{n,k}$ and $0{}_{<}^{>}-\gamma_{n}\theta_{j_{k},n}+\Lambda_{n}\left(\kappa \right)$, over all *n* = 1, …, *N* is called a *parameter cell*. The collection of all parameter cells decomposes parameter space.

For a network node *n* which has a single external input *S* and no other incoming edges from the network, we consider *n*-field inequalities $0{}_{<}^{>}-\gamma_{n}\theta_{j_{k},n}+S$. If node *n* has *k* out edges and hence *k*-thresholds $\theta_{n,1},\ldots ,\theta_{n,j_{k}}$ there are *k* + 1 such selections for each fixed threshold order given by *n*-threshold inequalities (1). Observe that for each parameter value in a given parameter cell the associated state transition graph will be identical, and therefore, to each parameter cell we can assign a unique Morse graph. The parameter graph is a graph where each vertex corresponds to a nonempty parameter cell. There is an edge between two vertices if and only if all but one of the inequalities that define the corresponding parameter cells are the same.

The parameter graph is always a product of parameter graphs associated to each vertex of the regulatory network. To see this observe that the choice of an instantiation of *n*-threshold inequalities is independent on the choice of an instantiation of *k*-threshold inequalities for *n* ≠ *k*, and therefore the set of all such instantiations decomposes as a product

$$\begin{array}{c} PG=\prod_{n=1}^{N}PG\left(n\right) \end{array}$$ (4)

where *N* is the number of vertices in the regulatory network. However, it is important to realize that the choices of *n*-threshold inequalities are not independent of each other when there are multiple thresholds (associated with out edges vertex *n*) and/or multiple in edges. To describe the form of *PG*(*n*) for vertices with more than one input and/or one output requires additional notation.

Given a vertex *v*_*n*_ in a regulatory network let *S*_*n*_ > 0 be number of inputs (in-edges) and *T*_*n*_ > 0 be the number of outputs (out-edges). Every edge *v*_*j*_ → *v*_*n*_ has three parameters, *l*_*nj*_, *u*_*nj*_, and *θ*_*nj*_. Therefore, there are $2^{S_{n}}$ possible *input values* to vertex *v*_*n*_:

$$\begin{array}{c} \left\{l_{nj_{1}},u_{nj_{1}}\right\}\times \cdots \times \left\{l_{nj_{S_{n}}},u_{nj_{S_{n}}}\right\} \end{array}$$

that we represent by binary numbers $In_{n}≔{\left\{0,1\right\}}^{S_{n}}$ under the mapping $l_{nj_{k}}\to 0$, $u_{nj_{k}}\to 1$. We view *In*_*n*_ as a partially ordered set where the order is inherited from the embedding of *In*_*n*_ to $R^{S_{n}}$.

We define

$$\begin{array}{c} PG\left(\neg j\right)≔\{G=\nu \times PG\left(j\right)|\nu \;\text{a}\;\text{vertex}\;\text{in}\;\prod_{i=1,i\neq j}^{n}PG\left(i\right)\}. \end{array}$$

The collection of out-edges from *v*_*i*_ correspond to a collection of thresholds $\Theta_{i}≔\left\{\theta_{l_{1},i},\theta_{l_{2},i},\ldots ,\theta_{l_{T_{i}},i}\right\}$. Every ordering *O* of the set Θ_*i*_ defines *T*_*i*_ + 1 *states* of *v*_*i*_:

$$\begin{array}{c} \left(0,\theta_{l_{1},i}\right),\left(\theta_{l_{1},i},\theta_{l_{2},i}\right),\ldots ,\left(\theta_{l_{T_{i}},i},\infty \right) \end{array}$$

that we label as the integers $U_{i}^{O}≔\left\{0,1,\ldots ,T_{i}\right\}$.

We say that a function $f:In_{i}\to U_{i}^{O}$ is *monotone* if *a* < *b* implies *f*(*a*) ≤ *f*(*b*).

The node of a parameter graph *PG*(*i*) is *ω*_*k*_ ≔ (*g*_*k*_, *O*_*k*_) where *O*_*k*_ is a particular order of thresholds Θ_*i*_ and *g*_*k*_ is a monotone map

$$\begin{array}{c} g_{k}:In_{i}\to U_{i}^{O_{k}}. \end{array}$$

Two nodes (*g*_*k*_, *O*_*k*_) and (*g*_*l*_, *O*_*l*_) in *PG*(*i*) are joined by an edge, if the orders *O*_*k*_ and *O*_*l*_ differ by transposition of exactly one inequality; or, if *O*_*k*_ = *O*_*l*_ and there is exactly one choice of *ζ* ∈ *In*_*i*_ such that |*g*_*k*_(*ζ*) − *g*_*l*_(*ζ*)| = 1. Note that these two choices correspond to a single difference in a choice of inequality in (1) and (2), respectively.

It is important to note that DSGRN database represents the dynamics not just of the network itself, but also the dynamics of all its subnetworks. This significantly increases the size of the parameter graph and we often chose to compute the *essential subgraph* of the parameter graph that only contains parameter nodes at which all network edges are *essential*.

Let

$$\begin{array}{c} C_{j}^{i}≔\left\{\left(b_{1},b_{2}\right)\in In_{i}\times In_{i}\;|\;b_{1}\;\text{and}\;b_{2}\;\text{differ}\;\text{in}\;\text{exactly}\;\text{the}\;j\text{-th}\;\text{coordinate}\right\}. \end{array}$$

**Definition 0.1.** Fix a network *RN*(*V*, *E*), parameter node *p* and an edge *v*_*j*_ → *v*_*i*_ ∈ *E* with associated threshold *θ*_*ij*_. Assume that *θ*_*ij*_ is between states *k* and *k* + 1 in *U*_*j*_. We say that the edge *v*_*j*_ → *v*_*i*_ is

1. *input-essential* if $\exists \left(b_{1},b_{2}\right)\in C_{j}^{i}$ such that *g*_*i*_(*b*_1_) ≠ *g*_*i*_(*b*_2_); and
2. *output-essential* if ∃*b*_1_,*b*_2_ ∈ *In*_*j*_ such that *g*_*j*_(*b*_1_) ≤ *k*, *g*_*j*_(*b*_2_) ≥ *k* + 1.

In other words, an edge *v*_*j*_ → *v*_*i*_ is input-essential if it provides a non-constant input to the target node *v*_*i*_, and an edge *v*_*j*_ → *v*_*i*_ output-essential if if some values of *In*_*j*_ drive *v*_*j*_ above *θ*_*ij*_ and some drive it below *θ*_*ij*_.

**Definition 0.2.** Fix a network *RN*(*V*, *E*). A parameter node *p* is *i* *-essential* if

1. every in-edge of *v*_*i*_ is input-essential; and
2. every out-edge of *v*_*i*_ is output-essential.

A parameter node *p* is *essential* if it is *i*-essential for all *i* = 1, …, *n*. The *essential parameter graph* is a subgraph of the parameter graph induced on essential parameter nodes.

### DSGRN database for E2F-Rb networks

The main biological phenotypes of the E2F-Rb network are quiescent state (QS) and proliferative state (PS). We have identified them in DSGRN as minimal nodes in Morse graphs with labels FP(*,…,*,0) and FP(*,…,*,m), for some *m* ≥ 1, respectively.

We now describe the more complicated phenotypes of resettable bistability and hysteresis that are function of the external input *S*.

### Resettable bistability and hysteresis in restriction point networks

As noted in (4) the parameter graph *PG* is a product of parameter graphs *PG*(*i*) that correspond to individual nodes *i* of the network. The input *S* enters the network at a particular node, which in E2F-Rb networks is either node MD, or the node Myc; for the START network it is the node representing Cln3. We call this the *receiving node* *R*. Increasing *S* correspond to a path in the parameter graph *PG*(*R*). As mentioned in the previous subsection, if *R* has *k* out edges, and no in-edges, the parameter graph *PG*(*R*) has *k* + 1 elements that are linearly ordered. Parameter graphs for *R* with multiple in- and out edges are much more complicated [8] but they always form a partially ordered set with the unique *lowest node* and the *highest node*.

The lowest parameter node in *PG*(*R*) is the parameter node where all outputs of the node *R* are below all thresholds of the nodes *R* connects to; this corresponds to the situation where *R* is sub-threshold to all of its outputs and so it is always off. The highest parameter node in *PG*(*R*) is the parameter node where all outputs of the node *R* are above all thresholds of the nodes *R* connects to; this corresponds to the situation where *R* is super-threshold to all of its outputs and so it is always on. We call an path in a parameter graph *PG*(*R*) a *full path* if starts at lowest parameter node and finishes at the highest parameter node.

A full path in *PG*(*R*) represents the effect of varying the input *S* through the entire response range of *R*: the input *S* moves *R* from being sub-threshold to being super-threshold with respect to all its downstream nodes.

Since the range of the input signal *S* does not have to be always matched to the range of *R*, we will also consider *partial paths* that are subpaths of the full paths. Note that these include constant paths. Constant paths correspond to input that has no measurable impact on the dynamics of the network. We will search for partial path hysteresis and resettable bistability, as well for full path hysteresis and resettable bistability. We perform these searches in the parameter graph

$$\begin{array}{c} PG≔PG(R)\times EPG(\neg R), \end{array}$$

where we take only essential parameter values for all non-receiving nodes, but allow non-essential parameter nodes for the receiving nodes of the network. Fix a node *G* in the parameter graph *EPG*(¬*R*); that is, we fix all parameter inequalities for all non-receiving nodes. To completely characterize a parameter node in the parameter graph, and hence the parameter domain in the parameter space, we need to select inequalities for the node *R*. For each such a choice of a node in *PG*(*R*) together with the fixed choice of a node in *EPG*(¬*R*), there is a well-defined Morse graph.

We search for resettable bistability in *G* by checking for the existence of a lifted path, either partial or full, in *G* that satisfies the properties listed in the Results section. In particular, for full path we check if the Morse graph in *G* at the lifted lowest node in *PG*(*R*) has a unique minimal node in the quiescent state QS. We then check that a Morse graph at least one other lifted node *B* is (QS, PS)-bistable. Finally, we check that there is at least one lifted path from *B* to lifted low node along which the Morse graph has either minimal node QS, or exhibits (QS, *) bistability.

To check for hysteresis in *G*, in addition to checking for resettable (QS, PS) bistability to QS, we also check for (QS, PS) bistability to PS, where *G* is required to have a unique minimal node in the proliferative state PS at the lifted high node of *PG*(*R*).

We make occasionally two modifications to this search. When the network node *R* that receives the input *S* has only single out-edge to a node *Q*, the node *R* only transmits the input information *S* to the rest of the network via *Q*. In such a situation, we search for bistability and resettable bistability in *PG*(*Q*) instead of *PG*(*R*). Since for networks considered in this paper, *S* always activates *R*, if the edge from *R* to *Q* is also activating, then we search for the resettable bistability and hysteresis in *PG*(*Q*) as described above for *PG*(*R*). However, if the edge from *R* to *Q* is repressing, then high value of input *S* causes a high level of repression and hence low value of expression of node *Q*, and low value of *S* causes high value of expression of the node *Q*. Therefore our search for resettable bistability and hysteresis in *PG*(*Q*) interchanges the roles of highest and lowest node of *PG*(*Q*) compared to their roles in *PG*(*R*). For example, for full path resettable bistability we check if the Morse graph in *G* at the lifted highest node in *PG*(*Q*) has a unique minimal node in the quiescent state QS. We then check that a Morse graph at least one other lifted node *B* is (QS, PS)-bistable. Finally, we check that there is at least one lifted path from *B* to lifted high node along which the Morse graph has either minimal node QS, or exhibits (QS, *) bistability. Similar modifications are made in search for hysteresis.

If there is multiple nodes in a row that only transmit the input *S*, for instance *S* → *R* → *Q* → *P* → … we repeat the above procedure and query hysteresis and resettable bistability along paths in *PG*(*W*) where *W* is the first node in the pathway which has either more than one input, or more than one output.

### Performance

The computations demonstrated in this paper can in principle be obtained by computing and subsequently querying a dynamical database for the network of interest. In particular, the database provides a lookup table allowing us to obtain the annotated Morse graph corresponding to each parameter graph node. We may use this lookup table as a subroutine as we search the subgraphs according to the algorithms in the previous section.

On the other hand, we found that this approach adds technical complexities to scaling to high performance computing. When scaling to many simultaneous cores, it becomes necessary to handle many database requests in parallel in an IO-efficient manner. While this appears to be a solvable technical problem, for our purposes it is easier to sidestep these complexities by not using a database, but rather computing the Morse graphs as needed. In other words, we simulate the database query by recomputing the Morse graph. This renders the majority of disk access unnecessary. With this approach, performing the searches described in the section Resettable bistability and hysteresis in restriction point networks, for each node in *PG*(*R*) is an example of an *embarassingly parallel* problem. That is, it can be trivially broken into pieces which may be solved in parallel. Our implementation exploits this and was run on a high performance computing cluster (see Table 1), which spread the roughly 10 million seconds of total compute time over approximately 800 cores, so results were available within a few hours [40]. We note that there is no obstacle to further scaling with this technique. It would also be possible to scale down: we could run these computations on a present-day laptop computer (using four CPU cores) in about a month.

**Table 1:** Computational time. CPU time to compute all four resettable bistability and hysteresis queries for various networks.

Network with edges:	time (s)
2a	21.87266
2a 2b	344.0229
2a 2b 7	224277.6
2a 2b 8	232487.9
2a 7	8638.32
2a 8	7733.29
2b	18.44617
2b 7	13359.98
2b 8	12681.72
7	546.7937
8	246.005
neither7 nor 8	3.467421
7 p27	4366.87
Yeast START	2.895762

### Code availability

In order to ensure our results may be reproduced we adhere to the following recipe: (1) We release our code under an open-source license, (2) We host our code on a publicly available site using version-control (i.e. history tracking), (3) We give the version numbers of the code used to produce the result, (4) We provide instructions for installing and running the code, and (5) We produce *digital object identifiers* (DOIs) of the versioned code for use in bibliographical entries. The computer codes used to reproduce the results in this paper are stored in two code repositories. The first repository is the DSGRN project [41]. This is an open-source project which, as of writing, is hosted on the code-sharing website GitHub at https://github.com/shaunharker/DSGRN. The version utilized for this paper is 1.0.0. The second repository is the supplemental for this paper [40] and houses the code (which relies on DSGRN) which is used to reproduce the above results. This again is open-source and is hosted at https://github.com/shaunharker/2017-DSGRN-IdentifyingRobustHysteresisInNetworks. The version utilized for this paper is 1.0.3. The DOIs for these can be found in the references.

## Supporting information

S1 Table Summary of all 3 node networks. We provide results for full path and partial path resettable bistablility and hysteresis for all 49 three node subnetworks. The first column contains the network number (referenced in Fig 3), second column a picture of the network, and columns 3-6 prevalence of partial path hysteresis, partial path resettable bistability, full path hysteresis and full path resettable bistability. The last column is time it took to complete the computation in each row.
(PDF)
