# Supplementary material for: Identifying robust hysteresis in networks
Source: PLoS Comput Biol. 2018 Apr 23;14(4):e1006121. doi: 10.1371/journal.pcbi.1006121 (PMC5933818; doi:10.1371/journal.pcbi.1006121)
Supplement: S1 Table — We provide results for full path and partial path resettable bistablility and hysteresis for all 49 three node subnetworks. The first column contains the network number (referenced in Fig 3), second column a picture of the network, and columns 3-6 prevalence of partial path hysteresis, partial path resettable bistability, full path hysteresis and full path resettable bistability. The last column is time it took to complete the computation in each row. (PDF) [file pcbi.1006121.s001.pdf]

network	figure	partial path hysteresis match	partial path resettable bistability match	full path hysteresis match	full path resettable bistability match	time
network00	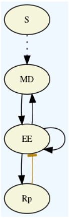	1.25362892584%	2.55344418052%	7.52177355503%	1.30641330166%	10.64239
network01	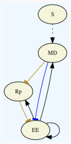	0.346313263525%	1.14028868528%	0.959860383944%	0.632635253054%	54.1616
network02	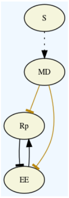	0.0%	0.0%	0.0%	0.0%	1.0004
network03	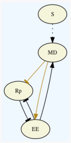	8.33333333333%	17.6339285714%	47.3214285714%	14.2857142857%	3.602331
network04	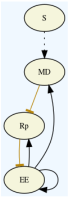	0.496031746032%	2.87698412698%	2.97619047619%	7.14285714286%	3.695112
network05	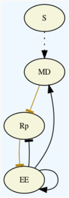	0.0%	8.33333333333%	0.0%	25.0%	2.943926
network06	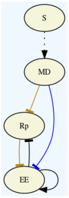	0.0%	3.75957854406%	0.0%	5.1724137931%	5.54425
network07	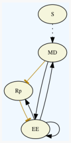	3.18603193455%	6.40010556875%	16.0876880443%	2.69200316706%	61.1844

network	figure	partial path hysteresis match	partial path resettable bistability match	full path hysteresis match	full path resettable bistability match	time
network08	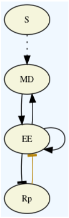	0.976510952758%	7.62734230668%	5.85906571655%	14.6080760095%	10.79685
network09	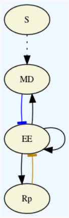	0.0%	0.123618382781%	0.0%	0.261780104712%	9.05131
network10	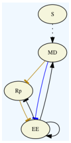	0.103621291449%	3.10500290867%	0.0%	4.01941535777%	57.8318
network11	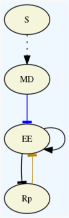	0.0%	0.0%	0.0%	0.0%	3.321078
network12	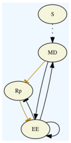	2.96829638427%	13.2266099235%	17.5969912906%	17.5178147268%	66.3699
network13	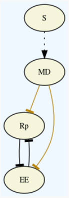	0.0%	4.16666666667%	0.0%	0.0%	2.596986
network14	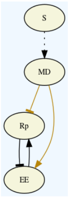	0.0%	0.0%	0.0%	0.0%	1.281634
network15	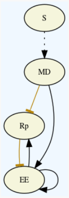	0.928442028986%	3.32880434783%	5.57065217391%	4.89130434783%	13.372057

network	figure	partial path hysteresis match	partial path resettable bistability match	full path hysteresis match	full path resettable bistability match	time
network16	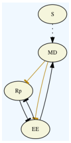	0.372023809524%	2.52976190476%	0.0%	1.78571428571%	13.47688
network17	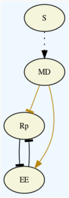	4.16666666667%	12.5%	25.0%	25.0%	12.42561
network18	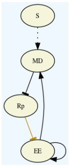	2.38095238095%	11.9047619048%	14.2857142857%	21.4285714286%	13.49131
network19	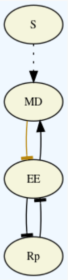	0.0%	0.0%	0.0%	0.0%	12.80522
network20	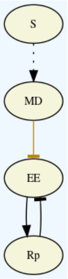	0.0%	0.0%	0.0%	0.0%	3.048265
network21	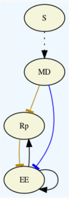	0.0%	1.41283524904%	0.0%	1.14942528736%	14.52947
network22	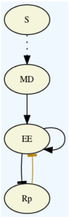	35.8490566038%	15.4986522911%	100.0%	0.0%	3.360046

network	figure	partial path hysteresis match	partial path resettable bistability match	full path hysteresis match	full path resettable bistability match	time
network23	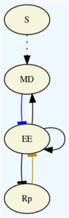	0.0%	0.0%	0.0%	0.0%	16.78121
network24	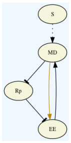	20.8333333333%	18.75%	100.0%	0.0%	0.593829
network25	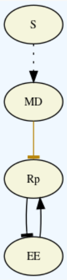	0.0%	0.0%	0.0%	0.0%	2.80243
network26	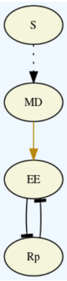	16.6666666667%	16.6666666667%	100.0%	0.0%	3.212279
network27	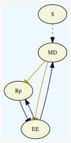	0.818452380952%	0.818452380952%	2.67857142857%	0.0%	2.122435
network28	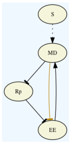	2.08333333333%	2.08333333333%	0.0%	0.0%	0.881725
network29	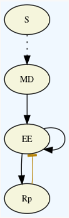	2.22371967655%	0.673854447439%	4.16666666667%	0.0%	3.496992

network	figure	partial path hysteresis match	partial path resettable bistability match	full path hysteresis match	full path resettable bistability match	time
network30	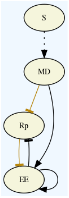	1.24547101449%	9.78260869565%	7.47282608696%	24.4565217391%	5.049274
network31	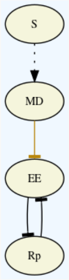	0.0%	0.0%	0.0%	0.0%	0.72931
network32	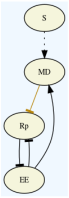	0.0%	16.6666666667%	0.0%	50.0%	13.92809
network33	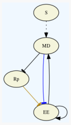	1.30507662835%	5.07662835249%	3.44827586207%	3.44827586207%	16.1358
network34	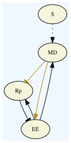	5.35714285714%	4.16666666667%	22.3214285714%	0.0%	14.93838
network35	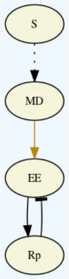	0.0%	0.0%	0.0%	0.0%	3.075829
network36	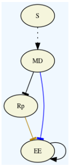	0.0%	5.55555555556%	0.0%	5.55555555556%	14.47661

network	figure	partial path hysteresis match	partial path resettable bistability match	full path hysteresis match	full path resettable bistability match	time
network38	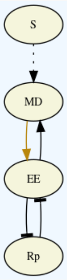	2.38095238095%	11.9047619048%	14.2857142857%	21.4285714286%	3.892062
network39	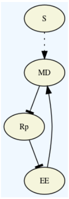	16.6666666667%	16.6666666667%	100.0%	0.0%	14.92302
network40	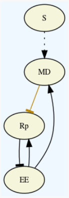	0.0%	0.0%	0.0%	0.0%	2.846478
network41	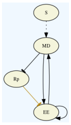	7.90307971014%	15.5910326087%	45.7880434783%	8.69565217391%	15.51011
network42	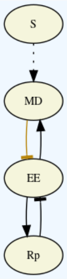	0.0%	0.0%	0.0%	0.0%	14.241749
network43	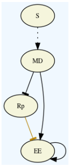	5.55555555556%	12.962962963%	33.3333333333%	22.2222222222%	15.00415
network44	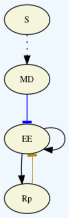	0.0%	0.0%	0.0%	0.0%	3.722113

network	figure	partial path hysteresis match	partial path resettable bistability match	full path hysteresis match	full path resettable bistability match	time
network45	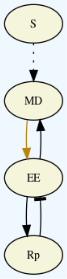	0.0%	0.0%	0.0%	0.0%	14.75178
network46	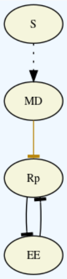	16.6666666667%	16.6666666667%	100.0%	0.0%	2.571777
network47	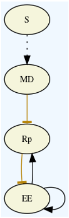	0.0%	7.14285714286%	0.0%	21.4285714286%	2.199021
network48	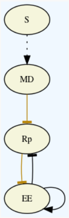	2.38095238095%	14.2857142857%	14.2857142857%	35.7142857143%	3.204714
